# Abdominal Aortic Aneurysm Repair: Results from a Series of Young Patients

**DOI:** 10.1155/2016/7893413

**Published:** 2016-09-29

**Authors:** Pasqualino Sirignano, Francesco Speziale, Nunzio Montelione, Chiara Pranteda, Giuseppe Galzerano, Wassim Mansour, Enrico Sbarigia, Carlo Setacci

**Affiliations:** ^1^Vascular and Endovascular Surgery Unit, Department of Surgery “Paride Stefanini”, Policlinico Umberto I, “Sapienza” University of Rome, 00161 Rome, Italy; ^2^Vascular and Endovascular Surgery Unit, Department of Medicine Surgery and Neurological Sciences, Policlinico Le Scotte, University of Siena, 53100 Siena, Italy

## Abstract

*Objectives.* To compare durability and survival after endovascular aneurysm repair (EVAR) and open repair (OR) of abdominal aortic aneurysms (AAAs) in young patients.* Material and Methods.* A retrospective study was conducted between 2005 and 2014 on all consecutive patients of 60 years of age or younger. Measures considered for analysis were reintervention related to AAA, laparotomy and access vessel injury during EVAR, and all-cause mortality during hospitalization and follow-up.* Results.* Seventy out of 119 patients were treated by OR (58.8%) and 49 (41.2%) by EVAR, 9 in off-label fashion (18.3%). Technical success was achieved in all cases. No AAA-related death was recorded. Overall in-hospital mortality was zero and the reintervention rate was 2.5% (3/119: 1/70 OR, 2/49 EVAR, *p* = 0.36). There is no death at 30-day or 1-year follow-up. Thirty-day reintervention rate was 1.6% (2/119; 0/70 OR, 2/49 EVAR, *p* = 0.16), while the 1-year rate was 2.5% (3/119; 1/70 OR, 2/49 EVAR, *p* = 0.36). At the mean follow-up of 56.8 ± 42.7 months, mortality and reintervention rates were 5.8% (7/119; 3/70 OR, 4/49 EVAR, *p* = 0.38) and 10% (12/119; 8/70 OR, 4/49 EVAR, *p* = 0.39), respectively. The overall reintervention rate, mortality, and freedom from adverse events did not differ between the two groups. No differences in outcome were recorded between patients treated by EVAR in on-label versus off-label fashion.* Conclusion.* Our (albeit limited) experience suggests that, in an unselected young patient population undergoing elective AAA repair, OR or EVAR can be performed safely with similar immediate and long term outcomes.

## 1. Introduction

Endovascular repair (EVAR) of abdominal aortic aneurysms (AAAs) was introduced by Parodi et al. in the early 1990s [1] as an alternative to conservative treatment in patients unfit for open repair (OR) [2]. Year after year, the role of EVAR has grown and now accounts for 80% of all AAA repairs performed in the USA [3]. EVAR is currently accepted as the procedure of choice for patients with AAA and suitable aortic anatomy [4], even in the absence of guideline recommendations. Moreover, as stent-graft design has improved and clinicians have become more skilled in stent-grafting techniques, standard stent-grafts have been implanted for more complex aortic anatomies, also outside the instructions for use (IFU), with acceptable early and long-term results [5–7]. Nevertheless, except for the OVER trial, large randomized controlled trials (RCTs) have only detected a clear benefit of EVAR with respect to OR in the early period, without further differences during follow-up [8–13]. This loss of late benefit has been ascribed to a higher incidence of reinterventions after EVAR, even if the great majority of such procedures were catheter-based operations associated with low mortality [14].

Loss of early benefit and the supposedly higher rate of reintervention have made physicians reluctant to use the endovascular solution in suitable young patients [15], although at the present time the very limited number of long-term studies on EVAR in young patients offers conflicting results and no level I evidence about the preferred method for this cohort of patients [2, 16–20].

This lack of evidence is difficult to understand because treatment of young AAA patients is a huge problem with more than 5000 procedures performed annually in the USA in patients between 50 and 64 years of age [21].

The aim of the present study was to review our experience on early and long-term outcome of young unselected patients (60 years of age or younger) electively treated for AAA in two high-volume Italian tertiary referral centres: the Vascular and Endovascular Surgery Unit at University of Rome “La Sapienza” and the Vascular and Endovascular Surgery Unit at the University of Siena.

## 2. Material and Methods

### 2.1. Study Design and Selection of Patients

A retrospective study was conducted on a prospectively compiled computerized database of consecutive AAA patients, 60 years of age or younger, between January 2005 and December 2014. Patients treated in urgent or emergency settings in the same period were excluded from the analysis, as were patients with mycotic AAA or aortic pseudoaneurysms. Indication for repair of AAA was based primarily on aneurysm diameter; speed of growth >1 cm/year and aortic wall morphology were also considered [22].

The choice between OR and EVAR was based on evaluation by surgeons, anaesthesiologists, and patients, considering patient status and comorbidities, AAA morphological features, relative benefits of one technique with respect to the other, and patient preference.

Local Ethical Committees were notified about the present study. Informed consent for aneurysm repair and participation in surveillance protocols was obtained from all patients.

All patients in the present series underwent preoperative contrast-enhanced computed tomography (CT). All images were reviewed retrospectively by two vascular surgeons. AAA morphology, including diameter, length, and angles, was assessed by OsiriX MD (OsiriX software; PIXMEO, Bernex, Switzerland) on a regular Mac OS computer [23]. As suggested by Lee et al. [18], a composite list of IFU criteria was used to define on- and off-label use of endografts: suprarenal aortic angulation <60 degrees, infrarenal aortic neck angulation <75 degrees, aneurysm infrarenal neck length >15 mm and diameter between 18 and 32 mm, and common iliac artery (CIA) distal fixation length >10 mm and diameter between 8 and 25 mm.

### 2.2. Intraoperative Details

All procedures were performed by vascular surgeons. For EVAR, inguinal field block [24] was used in 23/49 (46.9%) patients and all other cases were performed under general anesthesia. Bilateral surgical cut-down to the groin was performed in 31 patients (63.2%), while in 18 patients (36.8%) a percutaneous approach was used. Haemostasis was achieved using Perclose Prostar XL or Proglide devices (Abbott Vascular, Redwood City, CA, USA). Different commercially available stent-graft devices were used in this study, as shown in Table 1.

OR was performed by incision from the xyphoid to the pubis, transperitoneal approach, and infrarenal clamp in all patients. Reconstruction was performed by interposition of a straight graft in 39 patients (55.7%) and a bifurcated graft in 31 (44.3%). In all cases dacron grafts (Intergard, Maquet Getinge Group, Rastatt Germany) were used.

### 2.3. Endpoints and Definitions

Outcome measures were procedure-related reintervention and all-cause mortality rates during hospitalization and at 30-day, 1-year, and long-term follow-up. The reinterventions included in the analysis were related to aneurysm rupture, anastomotic pseudoaneurysm, graft infection, type I or III endoleaks, type II endoleaks with sac enlargement > 5 mm, graft stenosis or occlusion, and procedures related to renal or visceral ischemia. Reintervention related to laparotomy, such as lysis of adhesions or repair of abdominal wall hernia, was also considered, as well as reintervention for access vessel injury during EVAR. AAA-related and all-cause deaths were included in the analysis.

### 2.4. Follow-Up

In both centres the follow-up protocol included physical examination, duplex-ultrasound scan (DUS), and CT at 30 days. DUS was then performed at 3 and 6 months, at 1 year, and yearly thereafter. All patients underwent CT one year after the index procedure, without further CT examinations in the absence of complications detected by DUS [22, 25].

### 2.5. Statistical Analysis

The data is reported as means and standard deviations (SD) or as absolute frequencies and percentages (%). Intergroup comparisons for each variable were performed using Student's *t*-test for continuous variables and the *χ* ^2^-test or Fisher's exact test for categorical variables. A *p* value < 0.05 was considered statistically significant. Long-term survival and freedom from reintervention were determined by life-table analysis, Kaplan-Meier curves, and log-rank tests.

## 3. Results

One hundred and fifteen out of 119 patients were male (96.6%); mean age was 56.6 ± 3.5 years. Seventy patients were treated by OR (58.8%) and 49 (41.2%) by EVAR. There was no statistically significant difference in demographic or clinical characteristics between the two groups, although EVAR patients showed a higher frequency of dyslipidaemia and history of tobacco abuse (Table 2).

Preoperative CT showed mean maximum aortic diameters of 55.36 ± 13.2 mm in the OR and 54.45 ± 12.8 mm in the EVAR group. Patients' anatomical details are reported in Table 3, and no significant differences were found between the two study groups.

According to the composite list of IFU criteria, 9 out of 49 patients (18.3%) were treated in off-label fashion in the EVAR group. In detail, five patients had a 9 mm infrarenal neck, three patients had an infrarenal neck angulation between 75 and 85°, and one patient had both short and angulated neck. For the same morphological criteria, 17 out of 70 patients treated by OR (24.2%) had unfavorable anatomy: six had infrarenal neck length ≤9 mm, two had neck diameter >30 mm, five had suprarenal aortic angulation >75°, and four had narrow iliac access (diameter < 7 mm).

Technical success was achieved in all cases. No AAA-related death and no in-hospital mortality were recorded in the series. At 30-day follow-up, reintervention rates were 0 in the OR group and 4% in EVAR group (*p* = 0.16).

At the mean follow-up of 56.8 ± 42.7 months (range 12–120 months) mortality and reintervention rates were not different between the 2 study groups: *p* = 0.38 and *p* = 0.39, respectively. Details regarding mortality and reintervention during follow-up are reported in Table 4.

In detail, of the 10 reinterventions observed in the EVAR group, endograft explanation and conversion to OR were performed in two cases (one endograft infection after one year, and one type Ia endoleak 3 years after the index procedure). In the OR group, reinterventions were required for incisional hernia repair and lysis of adhesions, as well as one case of colon ischemia treated by left hemicolectomy and two cases of endovascular exclusion of anastomotic pseudoaneurysms. During follow-up of the present series, eight reinterventions were performed in the EVAR group due to endoleaks: five type Ia and three type Ib endoleaks. All high flow endoleaks requiring reintervention were detected during scheduled follow-up, and patients were electively treated. All but one type Ia endoleaks were managed with catheter-based procedures by a proximal aortic cuff implantation. Type Ib endoleaks were treated by limb extension into external iliac artery with intentional coverage of the ipsilateral hypogastric ostium. No type II leaks requiring reintervention were observed. Details of reintervention are shown in Table 5.

**Figure 1:**
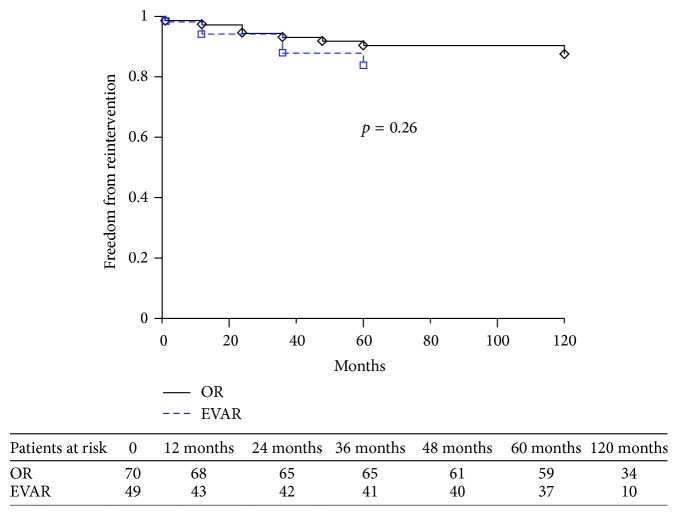
Kaplan-Meier estimates of freedom from reintervention; standard error never exceeded 10%.

**Figure 2:**
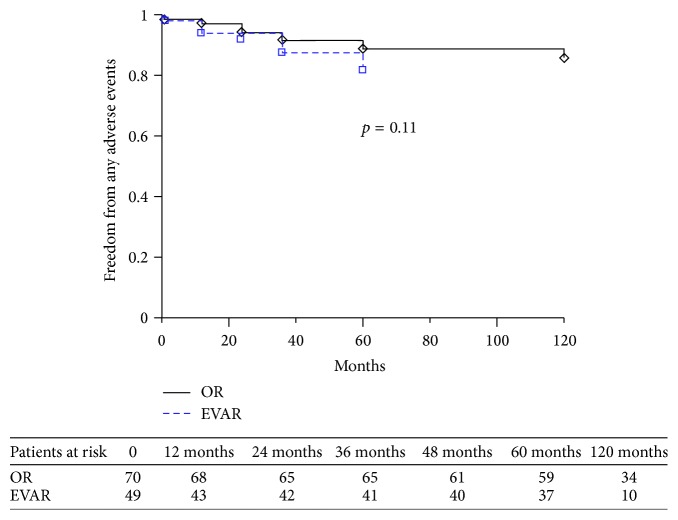
Kaplan-Meier estimates of freedom from any adverse event; standard error never exceeded 10%.

Univariate analysis and the log-rank test showed that rates of reintervention, mortality, and freedom from adverse events did not differ between the two groups: *p* = 0.26 (Figure 1), *p* = 0.21, and *p* = 0.11 (Figure 2), respectively. Even classifying incisional hernia repair and distal graft extension as minor procedures and excluding them from the analysis, no significant differences were observed (*p* = 0.10). No differences in outcome with regard to mortality (*p* = 0.42), reintervention (*p* = 0.59), and freedom from adverse events (*p* = 0.49) were recorded between patients treated by EVAR in on-label or off-label fashion.

## 4. Discussion

Since 1991 [1], the role of EVAR has grown year by year. Today it is the procedure of choice for patients with AAA and suitable aortic anatomy [3], as well as being a valid alternative in patients with challenging aortic anatomies [5–7]. Nevertheless, large RCTs have only shown a clear benefit of EVAR with respect to OR in the early period; this benefit was lost after three or four years of follow-up due to a higher rate of reintervention [8–13]. These concerns about long-term outcomes after EVAR have discouraged its use in younger subjects with long life expectancy [15, 26–29]. Moreover, among the four big RCTs comparing EVAR and OR, only the OVER trial [12] specifically examined results of OR and EVAR using a specific age criterion (<70 years). This means that no level I evidence exists about the preferred method for this cohort of patients. As a result, the most appropriate treatment modality for young patients suitable for OR is still debated [19, 20, 28, 29].

Our results show no significant differences in terms of early and long-term mortality and reintervention rates between young patients treated by EVAR and OR (*p* = 0.21 and *p* = 0.26). These findings are consistent with results reported by Altaf and Lee, showing no differences between the two types of treatment [16, 18]. Even a recent meta-analysis by Kontopodis et al. on more than 40,000 patients confirmed absence of any difference in outcome between OR and EVAR in subjects younger than 65 years of age, suggesting that EVAR should not be discouraged solely on the basis of age [19].

In addition, about 80% of reinterventions performed in the EVAR group were managed by catheter-based techniques. This is a significant difference with respect to other series. Lee et al. reported a small difference in the distribution of reinterventions after EVAR with 3 out of 7 patients undergoing catheter-based secondary procedures [18]. Altaf et al. reported a higher incidence of open reinterventions after EVAR, but this substantial difference could be due to the different time periods, since their series goes back to 1994 and reports results with first-generation devices over a longer follow-up period [16]. This large proportion of catheter-based reinterventions after EVAR could be important, since about 80% of reinterventions after OR in this series (even incisional hernia repair) required further open abdominal surgery.

In present experience we report a 20% of reintervention after EVAR at long-term follow-up, a result consistent with the 16% reintervention rate reported by Altaf et al. [16] in a similar cohort of patients. Of note, in both series no type II requiring reintervention was detected.

The main differences between our results and previous ones are regarding strict adherence to IFU. Lee et al. strongly recommend EVAR only in on-label situations, whereas in the present series encouraging results were also achieved in patients treated by EVAR in off-label settings [18]. In any case, only nine patients were treated outside the IFU, so we do not have sufficient data to make any kind of recommendation.

Those promising findings, associated with results published by Verzini et al. showing that outcomes after EVAR are continuously improving and that the early advantage of EVAR is maintained for at least 7 years, seem to support our strategy of offering less invasive treatment even to young patients [30].

Last criticism considering EVAR in young patients is represented by follow-up modalities. CT is considered the gold standard technique for follow-up after EVAR, although it raises several concerns for the required lifelong surveillance [22]. The cumulative dose of radiation administrated to patients and the use of intravenous contrast medium could potentially represent a disadvantage, especially in young subjects [31]. Therefore, the necessity to explore a different follow-up modality has prompted different authors to validate magnetic resonance [32], contrast-enhanced ultrasound [33], and digital tomosynthesis [25] as safer alternative to CTA. Widespread use of those surveillance modalities could overtake the last EVAR restraint.

The present study has several limitations. It is a retrospective study conducted on a relatively small cohort of patients. One major bias was the lack of randomization between the EVAR and OR groups. No propensity-scored matching analysis was performed. Moreover, all procedures were performed in the same centres and by the same skilled operators with equal experience in both kinds of repair; this could partially explain the small number of reinterventions recorded, as well as the fact that the most complex cases were presumably treated by open surgical repair. No centralized core-lab was used for preoperative CT analysis.

In conclusion, it is currently impossible to obtain definitive evidence about EVAR in patients younger than 60 years of age, but our preliminary results seem promising. Larger studies and longer follow-up are needed.

## Figures and Tables

**Table 1:** Commercially available stent-graft devices used in this study.

	49 patients	%
*Excluder and C3* (W. L. Gore & Associates, Flagstaff, AZ, USA)	17	34.7
*Talent* (Medtronic Inc., Santa Rosa, CA, USA)	11	22.5
*Endurant I and Endurant II* (Medtronic Inc., Santa Rosa, CA, USA)	10	20.4
*AFX* (Endologix, Irvine, CA, USA)	5	10.2
*Zenith* (Cook Medical, Bloomington, IN, USA)	2	4.1
*Nellix* (Endologix, Irvine, CA, USA)	2	4.1
*Ovation* (Endologix, Irvine, CA, USA)	1	2
*Treovance* (Bolton Medical, Sunrise, FL, USA)	1	2

**Table 2:** Demographic characteristics of patients included in this series.

	70 OR patients	49 EVAR patients	*p*
Suprarenal aortic angulation	12.7 ± 9.1°	13.8° ± 8.9	0.82
Infrarenal aortic angulation	23.1° ± 22.3	27.9° ± 24.2	0.75
Aortic neck length	25.4 ± 11.9 mm	22 ± 3.5 mm	0.43
Aortic neck diameter	24 ± 13.4 mm	20 ± 1.7 mm	0.24
R-CIA diameter	14.8 ± 6.22 mm	12.9 ± 6.9 mm	0.74
R-CIA fixation length	15.2 ± 2.5 mm	14.3 ± 6.8 mm	0.81
L-CIA diameter	14.7 ± 6.10 mm	12.7 ± 7.6 mm	0.76
L-CIA fixation length	14.9 ± 2.9 mm	14.4 ± 6.13 mm	0.73

OR: open repair; EVAR: endovascular repair; CAD: coronary artery disease; COPD: chronic obstructive pulmonary disease; CRI: chronic renal insufficiency.

**Table 3:** Aneurisms' morphological characteristics of patients included in this series.

	70 OR patients	49 EVAR patients	*p*
Age (mean, SD)	56.0 (±3.251)	57.4 (±2.754)	—
Male sex (*n*, %)	68, 97.1	47, 95.9	0.35
Hypertension (*n*, %)	51, 72.8	29, 59.1	0.29
Dyslipidaemia (*n*, %)	29, 41.4	26, 53.1	0.06
Diabetes (*n*, %)	11, 15.7	6, 12.5	0.40
CAD (*n*, %)	22, 31.4	11, 22.4	0.19
Smoke (*n*, %)	56, 80	33, 67.3	0.08
COPD (*n*, %)	8, 11.4	7, 14.2	0.42
CRI (*n*, %)	12, 17.1	8, 16.3	0.56

OR: open repair; EVAR: endovascular repair; R-CIA: right common iliac artery; L-CIA: left common iliac artery.

**Table 4:** Early and long-term survival and reintervention rates in present series.

	70 OR patients (*n*, %)	49 EVAR patients (*n*, %)	*p*
In-hospital			
Reintervention	1, 1.4	2, 4	0.36
Mortality	0, 0	0, 0	NA
30-day			
Reintervention	0, 0	2, 4	0.16
Mortality	0, 0	0, 0	NA
1-year			
Reintervention	1, 1.4	2, 4	0.36
Mortality	0, 0	0, 0	NA
Long-term			
Reintervention	8, 11.4	4, 8.1	0.39
Mortality	3, 4.2	4, 8.1	0.38

OR: open repair; EVAR: endovascular repair.

**Table 5:** Details of reinterventions in patients included in this series.

	OR (10 reinterventions in 70 patients)	EVAR (10 reinterventions in 49 patients)
In-hospital	1 left hemicolectomy	1 proximal extension 1 iliac extension

30-day	—	2 proximal aortic extensions

1-year	1 incisional hernia repair	1 iliac extension 1 conversion to OR

Long-term	5 incisional hernia repairs 1 lysis of adhesions 2 EVAR	1 iliac extension 1 proximal aortic extension 1 conversion to OR 1 femorofemoral crossover

OR: open repair; EVAR: endovascular repair.
